# Supplementary figures and images for: Co-culturing Hyphomicrobium nitrativorans strain NL23 and Methylophaga nitratireducenticrescens strain JAM1 allows sustainable denitrifying activities under marine conditions
Source: PeerJ. 2021 Nov 1;9:e12424. doi: 10.7717/peerj.12424 (PMC8567858; doi:10.7717/peerj.12424)

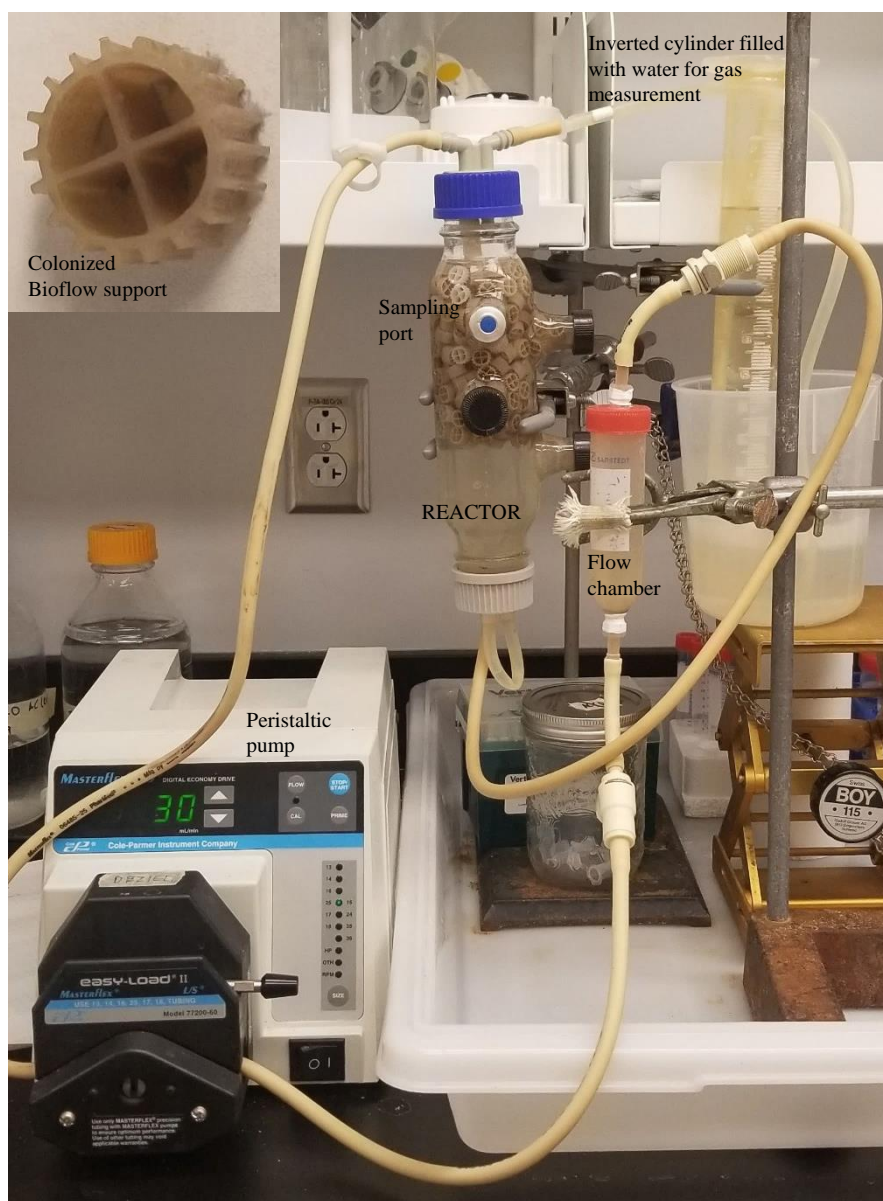

**Figure S1. Configuration of the reactor**

Supplement: Supplemental Information 1 [file peerj-09-12424-s001.pdf]
