# Supplementary material for: Co-culturing Hyphomicrobium nitrativorans strain NL23 and Methylophaga nitratireducenticrescens strain JAM1 allows sustainable denitrifying activities under marine conditions
Source: PeerJ. 2021 Nov 1;9:e12424. doi: 10.7717/peerj.12424 (PMC8567858; doi:10.7717/peerj.12424)
Supplement: Supplemental Information 2 [file peerj-09-12424-s002.docx]

Supplemental document

**Protocol for the measurement of the concentrations of NO_3_^-^ and NO_2_^-^**

Eight hundred mg VCl_3_ were dissolved in 100 mL of 0.1 M HCl (Solution A). The solution was filtered (0.45 µm). Solution B was made of 200 mg N-1-naphthylethylenediamine dihydrochloride in 100 mL H_2_O. Solution C was made of 2 g sulfanilamide in 100 mL HCl 10%. These solutions were kept in amber bottle at 4°C for one month. Solutions D and E were made fresh. For solution D, solutions A, B and C were mixed at the proportion of 5:1:1. For solution E, solutions B and C were mixed at 1:1 proportion. The assays were carried out in 96-well plates. To determine the NO_x_ concentrations (NO_3_^-^ + NO_2_^-^), samples (120 µL) were added to one plate, then 100-µL solution D was quickly added and mixed with a multichannel micropipet. The plate was covered and immediately incubated for 60 min at 45°C. To measure NO_2_^-^ already present in the medium, the same samples (200 µL) were added to another plate, then 20-µL solution E was quickly added and mixed with a multichannel micropipet. The plate was covered and immediately incubated for 30 min at 45°C. Both plates were read at 540 nm with a plate reader. The concentrations were determined with standard solutions. Linear response ranged from 0.1 to 2 mg-N/L (either NO_3_^-^ or NO_2_^-^). Results from the NO_3_^-^ reduction by VCl_3_ generated the NO_x_ concentrations (NO_3_^-^ + NO_2_^-^). Therefore, NO_3_^-^ concentrations were calculated as NO_x_ – NO_2_^-^.
