# Supplementary material for: Co-culturing Hyphomicrobium nitrativorans strain NL23 and Methylophaga nitratireducenticrescens strain JAM1 allows sustainable denitrifying activities under marine conditions
Source: PeerJ. 2021 Nov 1;9:e12424. doi: 10.7717/peerj.12424 (PMC8567858; doi:10.7717/peerj.12424)
Supplement: Supplemental Information 3 [file peerj-09-12424-s003.pptx]

## Slide 1
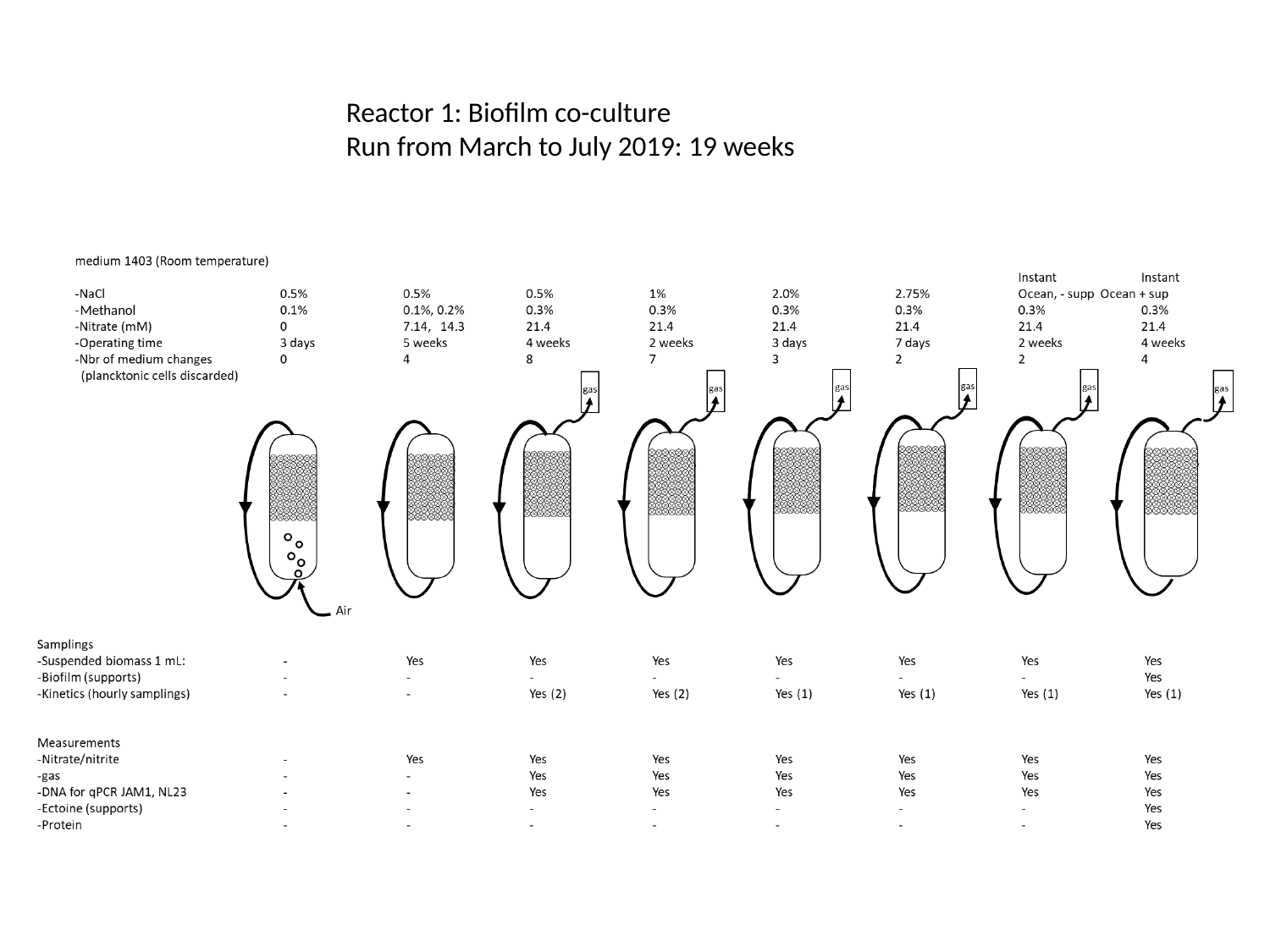

Reactor 1: Biofilm co-culture
Run from March to July 2019: 19 weeks
Methanol

## Slide 2
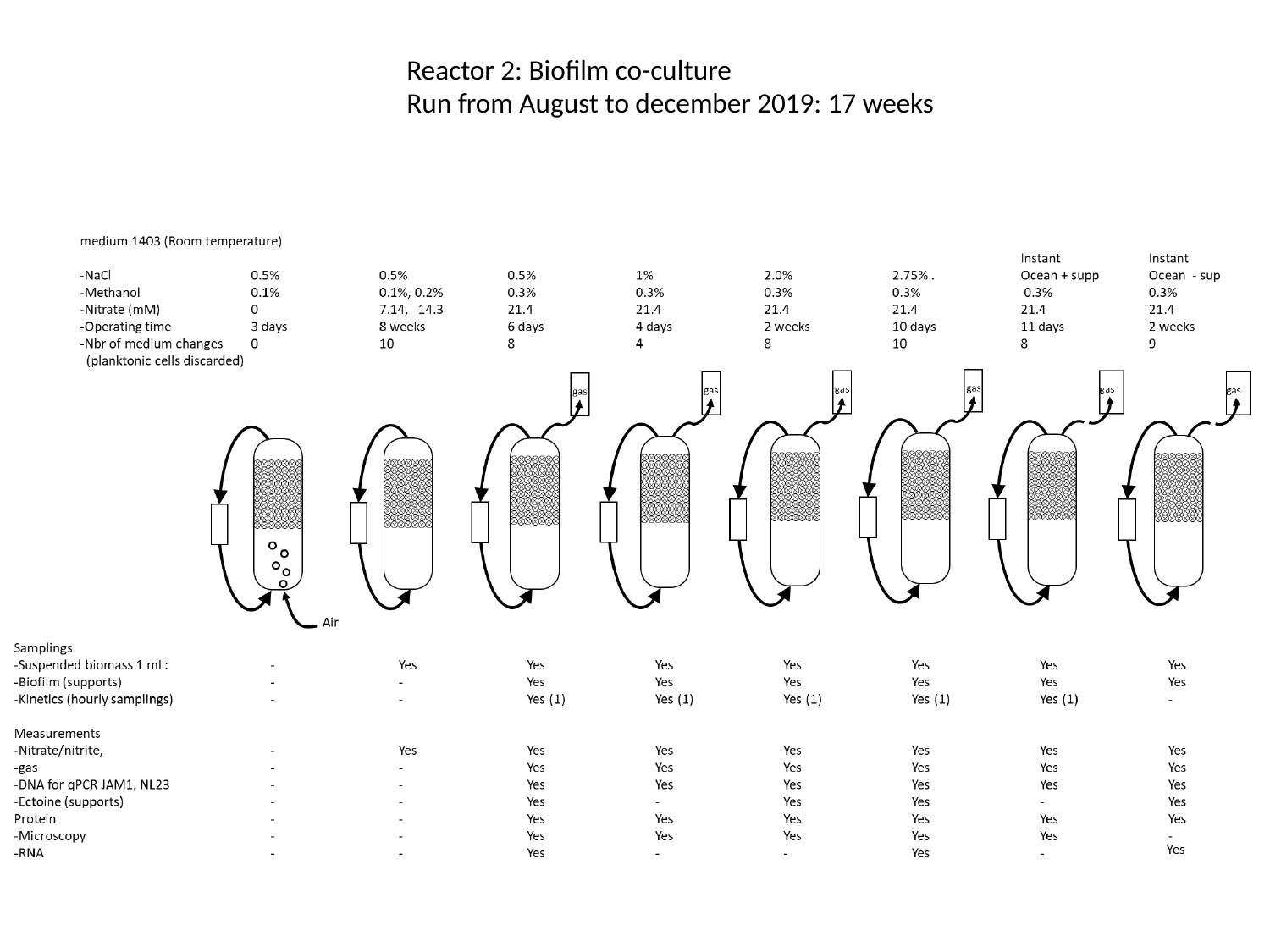

Reactor 2: Biofilm co-culture
Run from August to december 2019: 17 weeks
Yes

## Slide 3
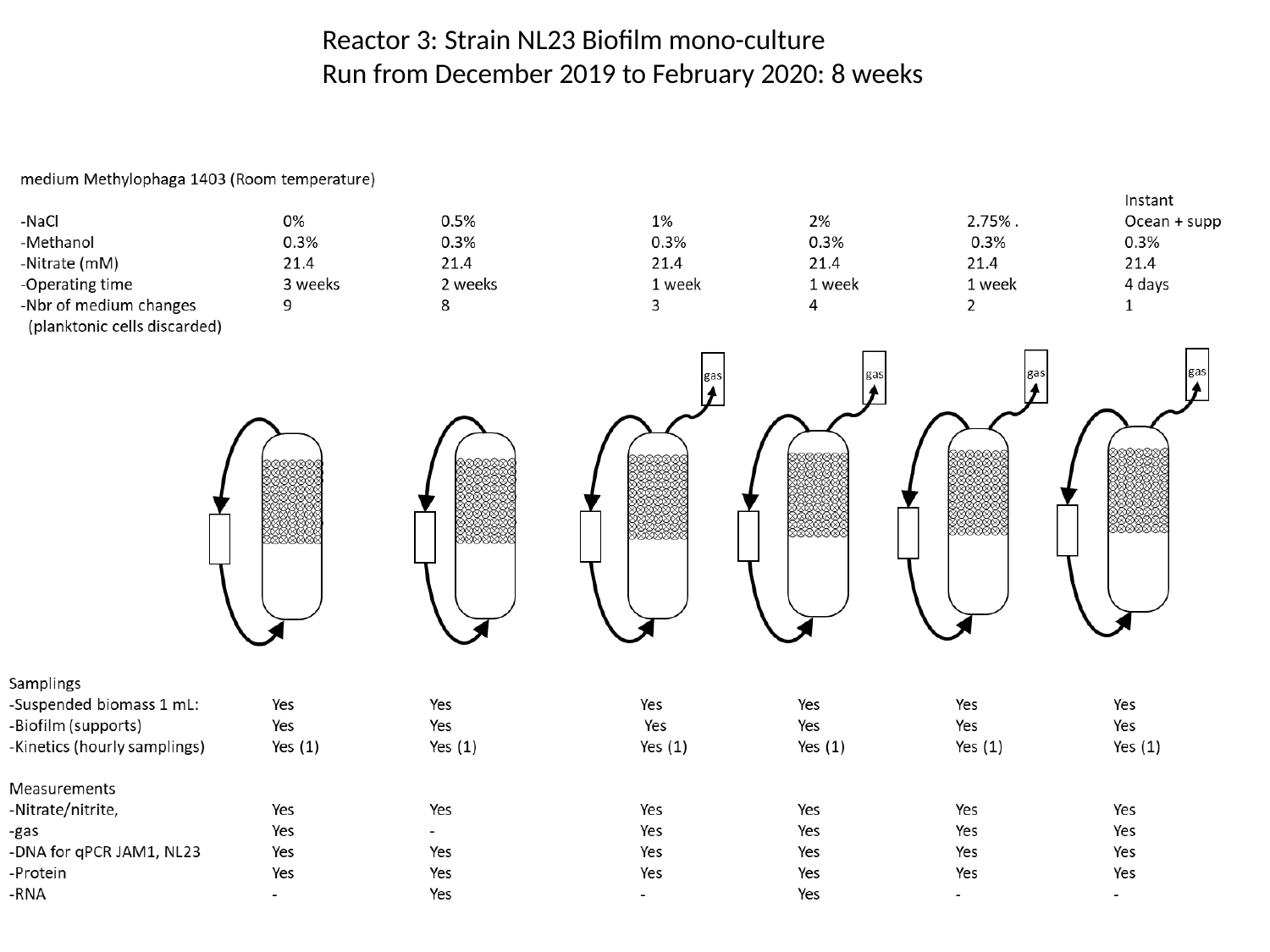

Reactor 3: Strain NL23 Biofilm mono-culture
Run from December 2019 to February 2020: 8 weeks
